# Cognitive Interviewing to Improve Infant and Young Child Dietary Assessments in the Nepal Demographic and Health Survey^☆^

**DOI:** 10.1016/j.cdnut.2024.104453

**Published:** 2024-08-30

**Authors:** Seema Khadka, Nisha Sharma, Katelyn Yuen-Esco, Alissa M Pries

**Affiliations:** 1 Helen Keller International Nepal, Lalitpur, Nepal; 2 Helen Keller International, NY, United States

**Keywords:** cognitive interviewing, cognitive validity, dietary assessment, infant and young child feeding, Demographic and Health Survey, complementary feeding indicators, survey measurement

## Abstract

**Background:**

The Demographic and Health Survey (DHS) is the primary source of nationally representative nutrition data for many countries and is important in shaping national nutrition policy, advocacy, and research. Without careful testing and adaptation to local contexts, findings are at risk of inaccurately representing respondents’ behaviors and key nutritional indicators.

**Objectives:**

This study used a cognitive interviewing approach to assess mothers’ comprehension, retrieval, judgment, and response to the infant and young child feeding (IYCF) dietary assessment module questions in the Nepal DHS-8 Woman’s Questionnaire.

**Methods:**

A cross-sectional qualitative study was conducted in December 2021, using semistructured cognitive interviews, among 54 mothers of children 6–23 mo of age across 3 regions of Nepal. Interviews were analyzed to identify issues in participant understanding of questions, as well as cognitive challenges or benefits of a longer introduction to the IYCF dietary assessment module, as compared with the original shorter introduction and challenges or benefits to using closed-ended compared with open-ended questions.

**Results:**

Cognitive challenges were identified, including issues with judgment of certain food categories resulting in misclassification, comprehension of certain concepts, and comprehension of certain instructions for the dietary assessment that resulted in the variability of food and liquids recalled by mothers.

**Conclusions:**

These findings carry substantial implications for the measurement of the diet quality of infants and young children (IYC) in Nepal, and similar cognitive challenges may exist in other contexts. Although this current study is not able to answer the exact extent to which the accuracy and precision of IYCF indicators are affected by these cognitive challenges, these findings indicate that current measurements may be subject to over or underestimation and there is a need for further research in this area to understand the degree of measurement error related to these challenges.

## Introduction

Accurate measurement in national population-based surveys is essential for evidence-based development of nutrition policies and programs, and to monitor trends at the regional, national, and subnational levels. The Demographic and Health Survey (DHS) is the primary source of nationally representative nutrition data for many countries and is important in shaping nutrition policy, advocacy, and research. Without careful testing and adaptation to local contexts, findings used from surveys like the DHS to inform nutrition policy and programs are at risk of inaccurately representing respondents’ behaviors and key nutritional indicators. To mitigate this risk, one of the most widely used methods in survey development is cognitive interviewing. This is a research methodology used to qualitatively assess the cognitive match between a quantitative survey’s intent and the respondent’s interpretation and response [1], ultimately improving question design and reducing measurement error [2]. Discrepancies between how questions are administered and how questions are understood can occur at any one of the 4 stages of the cognitive process of comprehension, retrieval, judgment, and response [3]. Comprehension is the participant’s understanding of the content and meaning of key terms, for example, ensuring they understand that “whole grains” refer to grains that contain all essential parts of the grain kernel, such as brown rice or whole wheat bread. Retrieval evaluates the participant’s ability to recall the content needed from a specific time to answer the question, such as remembering what they ate or drank over the past week. Judgment determines areas where the participant may feel uncomfortable with content, for instance, when being asked about feeding their child certain foods groups considered to be unhealthy. Lastly, response determines whether the participant can easily respond in the manner the survey is performed, for example, being able to accurately select portion sizes that were served or consumed. Cognitive interviewing can identify potential sources of error and provide valuable insight into a participant’s understanding of survey questions [4].

Updated every 5 y, the DHS Program’s Model Questionnaires form the basis for the data that are collected in each country. Within these Model Questionnaires, the Woman’s Questionnaire is used to collect data on women of reproductive age and their children, including a module on infant and young child feeding (IYCF) practices that involves a dietary assessment among infants and young children below 24 mo of age. This IYCF dietary assessment module is the basis for countries’ calculation of key WHO/UNICEF IYCF indicators, including minimum dietary diversity, minimum meal frequency, and the recently developed indicators of unhealthy food and beverage consumption [5]. Such indicators are vital to assess diet quality during the critical complementary feeding period, being used to either chart progress over time or note areas of concern. For DHS-8, several new questions on consumption of unhealthy foods and beverages were added to the IYCF dietary assessment to allow calculation of the new WHO/UNICEF IYCF indicators of unhealthy diets, including sweet beverage consumption, sugary food/salty or fried food consumption, and 0 fruit or vegetable consumption. The IYCF dietary assessment questions for DHS-8 also used country-specific examples of sentinel foods, which were identified during key informant interviews for the development of the Diet Quality Questionnaire [6]. Given the limited evidence on the cognitive performance of national population-based surveys, and because of the importance of accurate measurement in these modules, in 2021, the DHS piloted a cognitive interviewing study in Uganda to assess participants’ cognition of questions in several nutrition-related modules, including the updated IYCF dietary assessment module. Both the new consumption questions and the new wording for pre-existing consumption questions were tested in this Uganda pilot cognitive interviewing study. Additionally, the pilot tested 2 methodological approaches to assess if they improved cognition and, therefore, the reliability of dietary data. First, an assessment of a new longer introduction to the IYCF dietary assessment was tested against a shorter original version. Testing this longer version evaluated if it resulted in improved cognition for respondents. Second, the use of open-ended compared with closed-ended questioning for certain food categories was also explored. The cognitive interviewing of these 2 approaches was intended to evaluate how participants understood the categories and the participant’s accuracy in reporting foods accordingly. As the DHS Program was taking up this pilot study in Uganda, other organizations were encouraged to conduct similar research in other country contexts.

This present study was conducted to address this evidence gap by assessing the cognitive performance of the DHS-8 IYCF module in a South Asian context. Diets among infants and young children in Nepal are concerning; the 2016 Nepal DHS found that only 47% of children 6–23 mo of age consumed 4 or more food groups each day [7] and consumption of commercially produced unhealthy foods and sugar-sweetened beverages is highly prevalent [8]. The ability of policymakers and programmers to develop appropriate responses and to track improvements in these diets is heavily dependent on the accuracy of national data. The aim of this study was to improve the accuracy of reported dietary recall among mothers of children aged 6–23 mo, from a range of geographic settings and socioeconomic backgrounds, through cognitive testing of Nepal DHS-8 questions. The study used a cognitive interviewing approach to meet the following objectives: *1*) To assess mothers’ comprehension, retrieval, judgment, and response to the IYCF dietary assessment module questions in the Nepal DHS-8 Woman’s Questionnaire; *2*) to assess any cognitive challenges or benefits of a longer introduction to the IYCF dietary assessment module, as compared with the original shorter introduction; and *3*) to assess any cognitive challenges or benefits to using closed-ended compared with open-ended questions in the IYCF dietary assessment module.

## Methods

### Study design

Cognitive testing of the IYCF dietary assessment module from the Nepal DHS-8 Model Woman’s Questionnaire was performed for this study. In December 2021, a cross-sectional qualitative study was conducted, using semistructured cognitive interviews. The DHS IYCF dietary assessment module questionnaire was first administered to participants, and then participants were led through the cognitive interview to gather data on their comprehension, retrieval, judgment, and response. The DHS Program Nutrition Cognitive Testing Protocol was adapted for this Nepal-specific study [9]. Ethical approval for this study was provided by the Nepal Health Research Council (Registration no: 547/2021 P). Written informed consent was obtained from participants before the interview. Participants received a small gift in appreciation for their time participating in the study.

The study required ∼2 d for data collection training, 2 researchers and 2 note-takers, 1 wk per region for data collection, 2 mo for data transcription, and 2 mo for data analysis and synthesis.

### Study population and sampling

Cognitive interviews were conducted in 3 purposively selected districts of Nepal–Kathmandu, Dhading, and Nawalparasi. These districts were selected because their populations represent 2 different regions – the Hills (Kathmandu and Dhading) and the Terai (Nawalparasi). In alignment with the DHS sampling criteria for the IYCF module, the study population of interest was mothers of reproductive age (15–49 y of age) with ≥1 young child 6–23 mo of age. Cognitive interviewing typically requires a sample size of 5–15 respondents within each population of interest to reach saturation [4,10]. Therefore, in each district, a sample of 5 women was purposively selected for each of the 4 combinations of methodological survey approaches tested – long compared with a short introduction and open- compared with closed-ended question – resulting in a sample of 20 women per region and a total of 60 women (Table 1). Within each district, 5 women were randomly allocated to 1 of 4 methodological testing groups: short DHS-8 introduction and open-ended food category questions (group A1), short introduction and closed-ended food category questions (group B1), long introduction and open-ended food category questions (group A2), and long introduction and closed-ended food category questions (group B2).

**TABLE 1:** Study groups and number of respondents.

Group	The type of question tested	Introduction tested	No. of mothers per group	No. of districts^1^	Total number of women
A1	Open-ended	Short	5	3	15
A2	Open-ended	Long	5	3	15
B1	Closed-ended	Short	5	3	15
B2	Closed-ended	Long	5	3	15

1 Kathmandu, Dhading, Nawalparasi.

### Participant identification and recruitment

Mothers were purposively sampled in all 3 districts based on anticipated socioeconomic status (SES) and recruitment was conducted through local community liaisons. Dhading and Nawalparasi districts are involved in the United States Agency for International Development-funded Suaahara Project, a project dedicated to improving the health and nutrition status of pregnant and lactating women and children under the age of 2. The Suaahara Project has district-wide coverage across both Dhading and Nawalparasi and field staff retain a list of beneficiaries with sociodemographic details of households. In coordination with Suaahara field staff, in Dhading and Nawalparasi, a convenience sampling method was used to select 20 mothers of children 6–23 mo of age based on geographical access from the district headquarters and across a range of low and medium/high SES, based on a list of Suaahara beneficiaries that included socioeconomic status information. In the Kathmandu district, 2 wards with anticipated low and medium/high SES populations, based on local research staff knowledge of the communities, were purposively selected. Female community health volunteers of those wards assisted to identify and recruit 10 mothers of children 6–23 mo of age in each of the 2 wards using a convenience sampling approach. Before recruitment in the study, COVID-19 screening questions were asked of the caregiver to identify any symptoms of COVID-19. Those with probable cases of COVID-19 were excluded from the study and advised to maintain self-isolation and good hygiene practices. No confirmed cases of COVID-19 were identified during screening. Exclusion criteria for this study included *1*) If the child 6–23 mon of age was severely ill, *2*) non-residence of the mother or child within Kathmandu Valley, Dhading, or Nawalparasi districts, *3*) the child had a congenital/physical malformation that inhibited feeding, *4*) the mother’s refusal to participate in the study, and *5*) a confirmed or probable COVID-19 case within the child’s household.

### Study tools

Study tools were adapted from a pilot study conducted in Uganda, including modifications of foods and food groups specific to the Nepalese context [9]. The tools were translated from English into Nepali, back-translated, and pretested before finalization. Pretesting was conducted among 5 mothers with a child 6–23 mo of age living in the Bhaktapur district of Kathmandu Valley who were all Nepali speaking and met the inclusion criteria of the study.

#### DHS IYCF dietary assessment module questionnaire

Following consent, participants first received the interviewer-administered questionnaire adapted from the Nepal DHS-8 Women’s Questionnaire. This included questions on sociodemographic data, including maternal education, maternal age, and child age, as well as the Equity Tool, a web-based app [11] to gather information on participant asset ownership to derive the participant’s wealth status. The questionnaire then included the IYCF dietary assessment module, where the mother was asked if her child consumed specific beverages and foods in the previous 24 h. Half of the mothers received the current (short) introduction to the IYCF dietary assessment module and half received a revised, longer version. These introductions differed in several aspects: *1*) the long introduction specified there would be “yes” and “no” questions while the short introduction did not; *2*) the long introduction instructed respondents to think about foods and liquids eaten at different times of the day to help trigger recall; and *3*) the longer introduction contained ellipses between sentences where the interviewer would pause, allowing the respondent time to think about foods and liquids eaten by her child at the different times during the past 24 h highlighted by the interviewer. The 2 introductions tested are shown in Table 2.

**TABLE 2:** Short compared with long introductions tested.

Short introduction	Long introduction
Prior to questions 636a–636k (liquids): Now I would like to ask you about liquids that (NAME) had yesterday during the day or at night. Please tell me about all drinks, whether (NAME) had them at home, or somewhere else. Prior to questions 637a–637u (foods): Now I would like to ask you about foods that (NAME) had yesterday during the day or at night. I am interested in foods your child ate whether at home or somewhere else. Please think about snacks and small meals as well as main meals. I will ask you about different foods, and I would like to know whether your child ate the food even if it was combined with other foods. Please do not answer “yes” for any food or ingredient only used in a small amount to add flavor to a dish.	Prior to questions 636a–636k (liquids): Now I would like to ask you some yes-or-no questions about liquids or foods that (NAME) had yesterday during the day or at night. Please tell me about all the drinks and foods, whether (NAME) had them at home, or somewhere else. First, I would like you to think about yesterday, from the time (NAME) woke up, through the night. Think about the first thing (he/she) drank or ate after waking up in the morning …and anything (he/she) had to drink or eat mid-morning …Think about any liquids or foods (he/she) had in the middle of the day, and in the afternoon …Think about any liquids or foods in the evening or late-night... and any other liquids or foods (he/she) may have had throughout the day or night. First, I am going to ask you about liquids. Please listen to the list of liquids and, if (NAME) drank any one of them, say yes. Prior to questions 637a–637u (foods): Now I would like to ask you some yes-or-no questions about foods that (NAME) had yesterday during the day or at night. I will ask you about different foods, and I would like to know whether your child ate the food even if it was combined with other foods. Please listen to the list of foods, and if (NAME) ate any one of them, say yes. Please do not answer “yes” for any food or ingredient only used in a small amount to add flavor to a dish.

The use of open-ended compared with closed-ended questioning for certain food categories was also explored through 2 versions of the IYCF dietary assessment module. Specifically, questions on children’s consumption of meat, other vegetables, fruits, and sweet foods used either a closed-ended approach (1–3 questions about certain sentinel foods only) or an open-ended approach (1 question where several examples of foods were provided but the participant was asked to recall “other foods” that they believed fit into the category). The open-ended questions are the format used in the existing questions in the Nepal DHS-8, whereas the closed-ended questions used in this study are those used in the Nepal Diet Quality Questionnaire for infants and young children [12]. Half the sample received the open-ended approach and the other half the closed-ended approach across the 4 methodological testing groups. The 2 versions of these questions are shown in Table 3.

**TABLE 3:** Open-ended compared with closed-ended questions tested.

Open-ended	Closed-ended
637f. Any other vegetables, such as tomato, cauliflower, cabbage, gourd, eggplant, or other vegetables?	637f(i). Tomato, cauliflower, cabbage, gourd, or eggplant? 637f(ii). Bitter gourd, bottle gourd, green pumpkin, lady finger, or radish?
637h. Any other fruits, such as apple, banana, guava, watermelon, mulberries, or other fruits?	637h(i). Apple, banana, avocado, watermelon, mulberries, amla, or guava? 637h(ii). Grapes, raisins, peaches, plums, pomegranate, Asian pear, or jackfruit?
637k. Any other meat, such as goat, mountain goat, lamb/sheep, pig, yak, or chicken?	637k(i). Goat, mountain goat, bheda, buffalo, or yak? 637k(ii). Local pig or hybrid pig? 637(iii). Chicken, duck, or pigeon?
637r. Any sweet foods such as cake, biscuits, cookies, jeri/jalebi, mithai, toffees, or ice cream?	637r(i). Cake, biscuits, cookies, donut, haluwa, or jeri/jalebi? 637r(ii). Mithai, kheer, chocolates, candies, toffees, or ice cream?

#### Cognitive interview tool

The cognitive interviewing tool used verbal probing consisting of prescripted retrospective questions. This method was selected because verbal probing is more systematic, elicits more specific information, and has a reduced respondent burden compared with the “thinking aloud” approach [13]. The cognitive interview guide was developed using the guiding principles of the cognitive process, which was designed to target one or more stages in the 4-stage cognitive model [14]. Examples of the types of questions used to target areas of the 4 stages of cognition assessed are shown in Table 4.

**TABLE 4:** Example of questions used to target areas of the 4-stage cognitive model.

Stage of cognition	Example questions
Comprehension	- What did you think that I meant when I said [X]? - I asked you [X]? What does the term [X] mean to you? - What does the word [X] mean to you? - What items come to mind? Name some foods you thought of based on these instructions. - What did you need to think about to be able to answer this question? - If a decision was made to [X], how was the decision made [X]? - How is the term “X” different from the term “X”? - How is the word “X” different from the word “X”?
Retrieval	- Many people find it difficult to recall activities done a long time ago. How do you remember that [X]? - How do you remember that [X]?
Judgment	- Why did you pick [X] for your answer? - Do you think that most people answer this question honestly?
Response	- Think of [another woman/mother with a young child in your community; woman in your community]. Do you think that other [women] you know may find it difficult to answer these questions for any reason?” If yes: Why do you think they may find it difficult? - Do you think some people may have trouble answering questions on [X]? Why? If yes: What can we do to make this question easier to understand? - BROAD: How easy or difficult was it to answer questions on [X]? Why? - SPECIFIC: How easy or difficult was this question to answer? Why? - How easy or difficult was it to answer that question? Why? - Is your answer among the response choices?

### Study procedures

Participants were interviewed individually in their homes and interviews were conducted in Nepali. Interviews began with the IYCF dietary assessment module and were followed by the cognitive interviewing guide. The cognitive interview consisted of a fixed set of questions designed to assess comprehension, retrieval, judgment, and response. Participants were prompted with repetition and probing if they did not understand the questions; however, they were not provided with descriptions or examples of food/food names, and terms were not clarified or described. The study aimed to assess participants' comprehension of the questions, understanding of key concepts and terms, and ability to recall information, and all participants were asked all questions. Interviews were conducted by the Nepalese Principal Investigator and Co-investigator who had prior experience in conducting IYCF dietary assessments and qualitative interviews. The interviews were audio-recorded, and notes were taken by trained research assistants. Both interviewers shared and reviewed each other’s work at the end of each day to ensure completeness and consistency. Any discrepancies in the administration of the questions were resolved in consensus between the 2 interviewers.

### Data management and analysis

The sample characteristics of the participants were analyzed using SPSS version 20. The results of the national wealth quintile assessment of participants were automatically calculated by Equity Tool. All cognitive interviews were transcribed verbatim by the research assistants in Nepali and translated into English. Verification of the transcribed and translated documents was done by a co-investigator. The translated transcripts were imported to NVivo 12 for coding. A deductive approach was followed for the analysis of the cognitive interviews. First, a list of codes based on cognitive challenges identified was developed by 2 investigators based on mutual consensus. Once the list of agreed-upon codes was developed, the 2 researchers independently coded 10% of the same interviews using the predefined codes. Following this, they engaged in discussions regarding their coding and the codes. Through these discussions, they developed a common understanding and resolved any misunderstandings or confusions, reaching a mutual consensus. Subsequently, the interviews were coded separately by each researcher. Coded data were analyzed using techniques from thematic analysis [15]. Both investigators discussed the analysis and wrote the findings. Further, data were interpreted to capture the intent and interpretation gap of each dietary question and suggested modifications to the IYCF dietary assessment module were developed.

## Results

A total of 60 participants were sampled; however, 4 participants were excluded as they did not meet the eligibility criteria. The final number of participants in each study group was: group A1 (Open-ended, short introduction) – 15 participants; group A2 (Open-ended, long introduction) – 12 participants; group B1 (closed-ended, short introduction) –14 participants; group B2 (close-ended, long introduction) –15 participants. Among all 56 participants, almost half were Brahmin/Chettri, 42.9% (*n* = 24) had obtained higher secondary level education or higher, and the majority (91.1%, *n* = 51) were in the top 2 quintiles of wealth when compared with national urban wealth quintiles (Table 5). Sixty-six percent of the children were male, and just over two-thirds were over 12 mo of age.

**TABLE 5:** Sociodemographic characteristics of participants (*n* = 56).

Characteristic	Value, % (*n*)
**Location**	
Kathmandu	30.4 (17)
Dhading	35.7 (20)
Nawalparasi	33.9 (19)
**Age group (y)**	
≤20	8.9 (5)
21–35	85.7 (48)
>35	5.4 (3)
**Ethnicity**	
Brahmin/Chhetri	44.6 (25)
Dalit	25.0 (14)
Advantaged Janajati	26.8 (15)
Disadvantaged Janajati	3.6 (2)
**Educational status**	
No formal education	5.3 (3)
Primary	14.3 (8)
Secondary	37.5 (21)
Higher secondary	28.6 (16)
Graduate	14.3 (8)
**Engaged in paid labor**	14.3 (8)
**National wealth quintile**	
Lowest	1.8 (1)
Middle	7.1 (4)
Fourth	42.9 (24)
Highest	48.2 (27)
**Sex of child, female**	33.9 (19)
**Age group of child (mo)**	
6–11	30.4 (17)
12–23	69.6 (39)

Four areas of cognitive error were identified during analysis: *1*) understanding key concepts and terms, *2*) misclassification of liquids and foods, *3*) understanding and memory of instructions, and *4*) the influence of social desirability bias. Additionally, findings related to the comparison of the long compared with short introductions and the closed-ended compared with open-ended questionnaires were identified. Findings are detailed below and summarized with potential solutions to identified issues in Table 6.

**TABLE 6:** Summary of findings and suggested revisions for IYCF dietary assessment module from DHS-8 Model Woman’s Questionnaire.

Question number	Question	Results by cognitive stage	Suggested solutions
	Now I would like to ask you about liquids that (NAME) had yesterday during the day or at night. Please tell me about all drinks, whether (NAME) had them at home, or somewhere else. Yesterday during the day or at night, did (NAME) drink:	The recall period was not understood to include yesterday during the nighttime by most of the participants (Retrieval/Judgment).	Translation in Nepali was revised to convey the meaning of “all through the night.”
WQ 636a	Plain water		
WQ 636b	Infant formulas such as Lactogen, Farex, and Nan?	No problems were identified.	No suggested revision.
WQ 636c	Fresh animal milk, tinned or powdered milk?	Most participants did not understand the concept of tinned milk (Comprehension). Several participants thought powdered milk referred to infant formula (Judgment).	- Remove “tinned” from this list. - Include examples of powdered milk such as “Everyday” and “Redcow” and explicitly mention that this does not include breast milk substitutes.
WQ 636c2	Was the milk a sweet or flavored type of milk?	Confusion and misunderstanding of the concept of “sweetened” and inclusion of flavors that are not sweet (Comprehension).	Refer to “sweet flavors” and provide an example of how milk could be sweetened.
WQ 636d	Lassi?	No problems were identified.	No suggested revision.
WQ 636d	If yes, was the lassi a sweet or flavored type of lassi?	No problems were identified.	No suggested revision.
WQ 636f	Horlicks, Viva, or Bournvita	Potential for some participants to not answer honestly if these items are perceived to not be good for children (Judgment/Social desirability bias).	Take into consideration during analysis.
WQ 636g	Fruit juice, fruit drinks such as Real, Frooti, or sugar cane juice?	Miscategorization of some fizzy drinks and energy drinks as fruit drinks (Comprehension/Judgment).	Have question WQ 636h precede this question so that fizzy drinks are asked first. This may reduce confusion between the 2 categories.
WQ 636h	Sweet-bottled drinks such as Coke, Fanta, Sprite, or energy drinks such as Red Bull?	Potential for some participants to not answer honestly if these items are perceived to not be good for children (Judgment/Social desirability bias).	Take into consideration during analysis.
WQ 636i	Chiya, coffee, or herbal drinks? If yes, was the drink sweetened?	No problems were identified.	No suggested revision.
WQ 636j	Clear broth or soup?		
WQ 636k	Any other liquids?	No problems were identified.	No suggested revision.
	Now I would like to ask you about foods that (NAME) had yesterday during the day or at night. I am interested in foods your child ate whether at home or somewhere else. Please think about snacks and small meals as well as main meals. I will ask you about different foods, and I would like to know whether your child ate the food even if it was combined with other foods. Please do not answer “yes” for any food or ingredient only used in a small amount to add flavor to a dish. Yesterday during the day or at night, did (NAME) eat:	- The recall period was not understood to include yesterday during the nighttime by most of the participants (Comprehension). - Participants could not conceptualize “small amount” and “for flavor” together, but instead understood these 2 phrases separately as either “in small amount“ or “to expose the child to more/new flavor” (Comprehension/Judgment).	- Revise the Nepali translation to convey “all through the night.” - Remove instructions, “Please do not answer “yes” for any food or ingredient only used in a small amount to add flavor to a dish.”
WQ 637a	Dahi?	No problems were identified.	No suggested revision.
WQ 637b	Rice, paratha, naan, roti, pau roti, makai, or dhido?		
WQ 637c	Carrots or ripe yellow pumpkin?		
WQ 637d	Potato, yam, wild yam, or white sweet potato?	No problems were identified.	No suggested revision.
WQ 637e	Saag, spinach, mustard greens, fennel greens, pumpkin shoots, taro leaves, or amaranth greens?	No problems were identified.	See the suggested revision for WQ637v.
WQ 637v	Gundruk, fenugreek greens, or broccoli?	These 3 food items conceptually come under different food groups in the Nepali context. Broccoli is considered *tarkari* (vegetable group), fenugreek greens as *saag* (leafy greens), and gundruk as a fermented food. Having all 3 in one question created confusion for the participants, particularly because *saag,* which covers all items in WQ 637e, was already asked (Comprehension/Judgment).	Remove this question and revise WQ637e as: “Saag, gundruk, or broccoli?”
WQ 637f	Any other vegetables, such as tomato, cauliflower, cabbage, gourd, eggplant, or other vegetables?	Participants mentioned different vegetables that came to mind when responding to this question. Participants miscategorized potato, broccoli, and green leafy vegetables in this question (Comprehension/Judgment).	Consider using a closed-ended approach for this question.
WQ 637g	Papayas, ripe mango, apricot, or persimmon?		
WQ 637h	Any other fruits, such as apple, banana, guava, watermelon, mulberries, or other fruits?	No problems were identified.	No suggested revision.
WQ 637i	Liver or organ meat?	No problems were identified.	No suggested revision.
WQ 637j	Sausages, ham, bacon, or canned meat?	Only several participants understood what sausage was, and almost all participants had no understanding of ham, bacon, or canned meat (Comprehension/Judgment).	Consider explaining these items just after the question is read to participants.
WQ 637k	Any other meat, such as, goat, mountain goat, lamb/sheep, pig, yak, or chicken?	Fish was misclassified into this category by a few participants (Comprehension/Judgment).	Consider a closed-ended style or move fish question (637m) before this meat question.
WQ 637l	Eggs?	No problems were identified.	No suggested revision.
WQ 637m	Fish or dried fish?	No problems were identified.	No suggested revision.
WQ 637n	Lito, jaulo, daal, chickpeas, beans, soybeans, or quanti?		
WQ 637o	Almonds, peanuts, cashews, pistachios, or walnuts?		
WQ 637p	Paneer or cheese?		
WQ 637r	Any sweet foods such as cake, biscuits, cookies, jeri/jalebi, mithai, toffees, or ice cream?	The long list of examples in this question confused the participants (Response).	Consider breaking into 2 closed-ended questions to reduce the length of the list. This could include one with packaged (cake, biscuits, cookies, chocolates [using this term rather than “toffees” as it covers all candies, lollies, chocolate, toffees, etc.] or ice cream) and one with the traditional items (haluwa, jeri/jalebi, mithai, kheer, donuts).
WQ 637s	Chips, Kurkure, Cheeseballs, Wai Wai, samosa, pakora, puri, or tareko khaja?	- The term *tareko khaja* confused because many items may be sauteed/pan-fried, such as sauteed vegetables, beaten rice, and chickpeas and these items may be misreported (Comprehension/Judgment). - Some food items in the question are brand-specific (that is, Wai Wai) so foods of the same type but different brands may not be reported by the participants (Comprehension/Judgment).	- Replace tareko khaja with specific, commonly consumed deep-fried items, such as “fried potatoes.” - Replace “Wai Wai” with “chowchow, such as Wai Wai or Rara.”

Questions highlighted in gray were not included in cognitive interviewing.

### Cognitive challenge 1: comprehension of liquids and foods

Significant issues in comprehension of certain liquids and foods were noted, particularly the concepts of “powdered milk,” “tinned milk,” “sweetened liquids,” “processed meats,” and “fruit juice/fruit drinks.” These challenges were evident in participants' responses, revealing misunderstandings and knowledge gaps that could impact the accuracy of dietary surveys.

#### Concepts of “powdered milk” and “tinned milk.”

In question Women’s Questionnaire (WQ) 636c, respondents are asked if their child consumed “fresh animal milk, tinned or powdered milk” on the previous day. Many participants were not clear on the concept of “powdered milk,” with 11 participants reporting that they did not know what it was. Almost a third of participants (*n* = 22) considered powdered milk to be Lactogen (a breast milk substitute). Those who correctly understood the concept of “powdered milk” described it as milk in powdered form, commonly used to make tea and *kheer* (rice pudding).

None of the participants correctly understood the concept of tinned milk. The majority of participants (*n* = 32) reported that they either had never heard of the term or that they did not know what tinned milk was. Commonly listed items that participants considered tinned milk were infant formula (*n* = 12), fresh animal milk that is sold door-to-door in a tin container (*n* = 5), and condensed milk used for making *kheer*. Two participants reported that tinned milk was powdered milk.

#### Concept of sweetened liquids

Among some participants, there was confusion about what “sweet or flavored” meant about certain beverages. When asked “was the milk a sweet or flavored type of milk?,” about a milk their child drank, the majority of participants reported “sweet or flavored milk” to be adding sugar to milk (*n* = 34). Adding malt/chocolate powder to beverages such as Horlicks, Bournvita, or Viva was also understood by participants as sweet or flavored milk (*n* = 10). However, 10 participants reported having no idea what “sweet or flavored milk” was. Additionally, though the intention of this question is to identify if sweetened milk was fed to the child, several participants reported the addition of items that may not have been sweet: powdered milk, Cerelac (infant cereal), and Lactogen (a breast milk substitute). Similarly, although most participants correctly understood the concept of “sweet or flavored lassi” (WQ 636d) and cited this as when sugar (*n* = 18), fruits (*n* = 13), or other sweet items were added to the drink, 7 participants reported that they did not understand the concept of “sweetened or flavored lassi.”

#### Concept of processed meats

There was substantial confusion around the concept of processed meats among almost all participants. Most participants (*n* = 32) did not know what “sausages, ham, bacon or canned meat” were when asked if their child consumed any of these items, the previous day (WQ637j). When asked to name other foods they thought of for this category, participants reported frozen meat (*n* = 8), nonprocessed meat dishes (*n* = 3), or any packaged meats (*n* = 1). Two participants misunderstood ”sausage” as “tomato sauce” due to the phonetic similarity between “sauce” and “sausage.” Although some participants reported knowing what sausage (*n* = 13) and canned meat (*n* = 9) were, they did not have a clear understanding of these food items when asked to explain further. Canned meat was referred to as frozen meat or reported to be canned fish. Given her unfamiliarity with the concept of processed meats, 1 participant suggested asking respondents “if they understood such meats” before asking whether their children were given these foods.

### Fruit juice/drinks compared with other drinks

When asked if their child drank “Fruit juice, fruit drinks such as Real, Frooti, or sugar cane juice?” in the previous day (WQ 636g), the majority of participants considered the terms “fruit juice” and “fruit drinks” to be interchangeable (*n* = 35). However, 5 participants incorrectly reported fizzy drinks and energy drinks, such as Coke/Pepsi and Red Bull, to be included in this category.

### Cognitive challenge 2: response error for liquids and foods

Misunderstandings around several food groups led to response errors and the misclassification of liquids and foods by participants, including challenges in classifying different types of vegetables and distinguishing sweet foods from sweet liquids. The results highlighted significant areas of confusion that could impact the accuracy of dietary data collection.

#### Misclassification of “other vegetables”

When asked if their child consumed “Any other vegetables, such as tomato, cauliflower, cabbage, gourd, eggplant, or other vegetables?,” miscategorization of food items reported as “other vegetables” was commonly observed. Half of the participants (*n* = 28) reported potatoes as a vegetable. Participants noted that this was because the question asks if other *tarkari* (vegetable food group) were consumed and potatoes are generally considered *tarkari* in Nepal, with potatoes commonly mixed into vegetable dishes.

*“Potatoes come under vegetable group.”* (27 y, Higher secondary education, Nawalparasi)

“*If the potato is mixed with peas, then potato is also counted as vegetable so, I would mention potato when asked about other vegetables also.”* (30 y, Higher secondary education, Dhading)

Additionally, participants reported classifying green leafy vegetables under the “other vegetables” food group when asked this question. Eighteen participants mentioned spinach, mustard greens, or other greens when asked to list examples of “other vegetables” despite being previously asked if their child consumed “saag, spinach, mustard greens, fennel greens, pumpkin shoots, taro leaves, or amaranth greens” in a previous question (WQ 637e). Like potatoes, these green leafy vegetables are also considered to be part of the *tarkari* (vegetable) food group.

#### Misclassification of sweet liquids as sweet foods

Participants were asked if their children consumed “Any sweet foods such as cake, biscuits, cookies, jeri/jalebi, mithai, toffees, or ice cream?” (WQ 637r). Several participants reported sweet beverages rather than sweet food items, including sweet tea (*n* = 4), juice (*n* = 3), and malted drinks (*n* = 3).

#### Misclassification of fried foods

Participants were asked if their children consumed “Chips, Kurkure, Cheeseballs, Wai Wai, samosa, pakora, puri, or tareko khaja?” (WQ 637s). This question is linked to the WHO IYCF indicator question measuring the consumption of unhealthy salty/fried foods and is specifically interested in deep-fried carbohydrate-based foods [5]. *Tareko khaja* refers to fried snacks and is included in this question to capture other deep-fried foods, however, there was confusion over what type of fried foods fell into this question. Specifically, participants assumed it included items that were pan-fried/sauteed and not solely items that were deep-fried. This included food items like pan-fried *momo* (dumplings) and *chowmein* (stir-fried noodles), as well as homemade food items that are sauteed but not deep-fried, such as sauteed potato or meat.

#### Confusion of saag types broken up across 2 questions

There was strong comprehension when participants were asked if their child consumed “saag, spinach, mustard greens, fennel greens, pumpkin shoots, taro leaves, or amaranth greens” (WQ 637e). The food items in this list all fall under the *saag* (leafy greens) food category in Nepal. However, the following question asked participants if their child consumed “gundruk, fenugreek greens, or broccoli” (WQ 637v), which confused each of the 3 items listed and fell under a different food category in Nepal. Fenugreek greens are considered *saag*, and were perceived to relate to the prior question, broccoli is considered *tarkari* (vegetable), and gundruk is a preserved form of *saag.* Although all 3 are considered dark green leafy vegetables, it was observed that participants were confused when this list was read out, particularly because the prior question already asked about various forms of *saag*.

### Cognitive challenge 3: understanding and memory of instructions

Cognitive challenges related to the understanding and memory of instructions were also identified among participants. The results highlighted difficulties in recalling specific time periods and understanding instructions about excluding certain foods, which could affect the accuracy of dietary assessments for infants and young children.

#### Recall the previous day or night

Nearly all participants (*n* = 54) reported that they recalled the foods and liquids that their children consumed the previous day, not other typical days. Only 2 participants reported not recalling yesterday specifically; these participants reported feeding the same foods to their children every day and therefore felt there was no difference in foods given yesterday or any other typical day.

Many participants (*n* = 44) reported thinking about the recall period from morning until the child went to bed and did not include it during the night after the child went to bed. One reason they reported was that their children do not wake up to eat anything, except breastmilk, after they go to bed.

*“He doesn't eat anything other than mother’s milk at night, so I didn’t think about nighttime.”* (36 y, Secondary education, Dhading district)

*“My child doesn’t wake up at night. So, I recalled till the child went to bed.”* (27 y, Higher secondary education, Nawalparasi district)

Upon review of the Nepali translation provided in the DHS questionnaire, it was noted that while “during the night” is understood in English to include throughout the nighttime the Nepali translation does not convey this same construct. *Rati*, instead translates to feeding in the evening/night until one goes to sleep. This could explain why some participants did not consider the time frame all through the night. Only 12 participants understood the recall period as including all through the night.

*“I was thinking the time from waking of child, morning, afternoon, evening and night as well...yes, 24 hours.”* (34 y, Graduate education, Dhading)

#### Memory and understanding of instruction to exclude certain foods

The majority of participants did not correctly remember the instructions to not include certain types of foods when recalling what their child consumed in the previous day and night. Only 4 participants reported remembering the instruction “not to include foods in small amount for flavor,” which meant not to include condiments used in the dishes. Others reported remembering either “not to include food in small amount” (*n* = 4) or “not to include foods for flavor” (*n* = 4).

Further, the understanding of the concept of a “small amount to add flavor” varied. Although 15 participants correctly understood the phrase to mean condiments used in small amounts for flavor, 10 participants reported not understanding the concept. Five participants understood the phrase to mean food given in small amounts to children, like *daalmoth*, dry fruits, and puffed rice. Twenty participants reported thinking this meant to not include foods fed to increase children’s taste exposures, mostly including packaged foods like cheeseballs and chips. Six participants reported understanding “small amount to add flavor” as the foods which are fed occasionally to diversify taste exposure, and which are therefore fed in small amounts at first. Several participants (*n* = 4) interpreted the instruction to mean that they should omit foods that were fed as snacks between meals.

*“I thought… of packed foods, like daalmoth, puffed rice. If I give him a small amount of daalmoth, he will (learn to) eat other foods.”* (22 y, Higher secondary education, Dhading district)

*“Foods that we give in a small amount, for tasting only, which are not given to make them full and satisfied..*.*”* (28 y, Secondary education, Dhading district)

*“You had asked not to mention food used for flavor only, so I thought it came among those foods and I didn’t mention it. He is fond of daalmoth, chowchow (*instant noodle)*, so I give him in small amounts.”* (22 y, Higher Secondary, Dhading district)

### Cognitive challenge 4: social desirability response to questions

The perceived potential for social desirability bias and resulting judgment error in participants’ responses to certain questions varied across food group categories. Forty-two participants believed that most people would be honest in their response to whether their child consumed a malted drink. Several participants (*n* = 5) considered malted drinks to be good for children, and they therefore thought that others would not lie about children’s consumption of these beverages. However, others (*n* = 9) were not sure how others would respond to this question and 5 participants thought that there might not be an honest answer from others who perceive that such drinks should not be given to children.

*“Some will tell, some won’t. If they know they shouldn’t feed such things, they won’t tell. If they feel that these things can be given to a child, they will tell.”* (27 y, Higher secondary education, Nawalparasi district)

Participants had differing opinions on whether others would answer honestly when asked if their child consumed fizzy drinks. Half of the participants (*n* = 33) thought that people would answer honestly while 11 participants reported that those who perceived these drinks to be harmful may not provide honest responses.

*“People may not answer honestly as they may fear being asked the reason for giving such drinks to the children.”* (28 y, Primary education, Nawalparasi district)

*“Some may think there are harmful chemicals in sweet-bottled drinks, and it can’t be fed, and if they say that they have fed then they might be scolded.”* (38 y, Higher secondary education, Kathmandu district)

### Comparing long compared with short introduction

When comparing participants’ reactions to the long compared with a short introduction to the dietary assessment, the long version did not appear to provide any additional benefit in terms of aiding recall of foods consumed by the children. Participants reported that the focus on ”yesterday” in both versions helped them to think of the foods given the previous day, rather than any other day. Only 2 participants who were administered the long introduction reported that emphasizing specific times from morning to evening made it easier for them to recall foods consumed by their children the previous day. Forty-five participants reported thinking of all foods and beverages, even in small quantities, consumed by their children the previous day. There was no notable difference in reporting consumption of foods consumed in small quantities in groups who either received the short compared with long introductions to the dietary assessment. Eleven participants who received either the short (*n* = 5) or long introduction (*n* = 6) reported missing food items consumed in small amounts during 24-h recall.

### Comparing closed-ended compared with open-ended questions

The effect of closed-ended compared with open-ended questions was specifically tested for 5 food groups: *1*) other vegetables, *2*) dark green leafy vegetables, *3*) fruits, *4*) meat, and *5*) sweet foods. In the open-ended questions, the term “any other” provided participants the opportunity to think about other similar foods. With this open-ended approach, the potential for participants to miscategorize food items was observed. For instance, participants considered potatoes as a vegetable and said they would report potatoes under the “other vegetables” group. Similarly, when asked if their child consumed “Any other meat, such as, goat, mountain goat, lamb/sheep, pig, yak, or chicken” (WQ 637k), several participants reported fish in this category as it falls under the *masu* (meat) food category in Nepal.

Conversely, the benefit of the open-ended questionnaire is that it allowed participants to report examples of additional food items not listed in the closed-ended questionnaire, potentially reducing the risk of underestimation of food category consumption. This was observed for some food categories. When asked if their child consumed “Any sweet foods such as cake, biscuits, cookies, jeri/jalebi, mithai, toffees, or ice cream?” (WQ 637r), half of the participants (*n* = 26) reporting being able to think of other similar sweet items their children consumed, including chocolate (*n* = 22), Chocofun (chocolate wafer treat) (*n* = 12), lollipop (= 8), *haluwa* (sweet dish made of semolina) (*n* = 6), *kheer* (*n* = 4), and honey (*n* = 3). Although a similar trend was noted when participants were asked to report further examples of other foods belonging to the same category as “chips, Kurkure, cheeseball, Wai Wai, samosa, pakora, puri, or tareko khaja,*”* a smaller number of participants were able to provide further examples.

As some food items in this question are brand-specific, this may have limited other examples considered by participants. For example, several participants reported being confused when asked to give examples of “Wai Wai” as this is a brand of instant noodle so no further examples existed. It was only when they were asked to give examples of other instant noodles did they named other examples and brands, including *Rara, Mayos,* and *Rumpum*. Citing “Wai Wai” or other branded foods in a closed-ended question could result in underreporting of certain foods. Overall, miscategorization errors with open-ended questions were more commonly observed than reports of additional foods that were outside the closed-ended questions.

## Discussion

This study is one of the first to employ cognitive interviewing to improve the measurement of IYCF indicators in large-scale, population-based surveys. Mothers’ comprehension, retrieval, judgment, and response to the IYCF dietary assessment module questions in the Nepal DHS-8 Woman’s Questionnaire were explored, and the effect of several methodological approaches to dietary assessment was compared. Cognitive challenges were identified, including issues with comprehension of certain concepts, judgment of certain food categories resulting in misclassification, and comprehension of certain instructions for the dietary assessment that resulted in variability of food and liquids recalled by mothers. These findings carry potentially substantial implications for the measurement of the diet quality of IYC in Nepal, and similar cognitive challenges may exist in other contexts.

Several previous studies have used cognitive interviewing to explore participant understanding of various aspects of DHS questionnaires, including infant and young child nutrition counseling [16] and antenatal counseling and reported practices [17], but this Nepal study is one of the first to explore participant understanding of questions for the IYC dietary assessment. The various cognitive challenges identified in this study hold potentially significant implications for the measurement validity of certain IYCF indicators, including those that indicate diet quality unhealthy food consumption, and other important measurements for dietary intake. First, as consumption of potatoes and tubers is highly prevalent among infants and young children in Nepal and one of the most commonly consumed food groups for this age group [7], the response error of potatoes as “other vegetables” by participants could substantially overestimate minimum dietary diversity estimates and underestimate estimates of zero fruit or vegetable consumption [5]. Further, the WHO indicator of unhealthy food consumption, a measure of either sweet food or salty/fried food consumption, may also be subject to measurement error. First, the brand-specific examples of salty/fried food items in the Nepal DHS-8 questionnaire could result in an underestimation of consumption. In addition, the use of the broad term *tareko khaja* (fried foods) could result in overestimation through the inclusion of sauteed foods that do not fall under the deep-fried unhealthy food construct for this indicator. Finally, confusion over the concept of “sweet or flavoured milk” and the inclusion of milk containing non-sweet ingredients could yield overestimations of the indicator of sweet beverage consumption in this context. These findings carry potentially substantial implications for the measurement of the diet quality of IYC in Nepal and potentially other contexts where similar cognitive challenges may exist.

Comparison of a closed-ended compared with open-ended approach for specific questions revealed cognitive issues related to judgment with open-ended versions and the importance of sentinel food item selection for closed-ended versions. Open-ended versions allowed for response error by participants for some food categories, whereas the closed-ended versions resulted in underreporting of certain foods when the example foods provided were brand specific. In addition to the potential to misclassify compared with omit certain foods, studies have found cons of open-ended questions when considering respondent burden. A study among American women of lower socioeconomic status who received an open recall 24-h dietary assessment and a closed-ended food frequency questionnaire found that participants did not like having to answer questions about foods they rarely or never ate and that this contributed to a longer time to complete the interview [18]. Conversely, other researchers have identified that open-ended questions can result in greater respondent fatigue [19], which can lead to nonresponse or misclassification by respondents [20]. In contrast, closed-ended questions have been found to result in higher reporting of certain foods, as compared with open recalls in 24-h dietary assessments, particularly for foods that are not regularly consumed such as fruit, snacks, and beverages [[21], [22], [23]]. It has therefore been theorized that closed-ended questions could aid memory and result in more accurate estimations [24]. This present study in Nepal also found that the instructions to not report foods “used in a small amount to add flavor to a dish” were not remembered or misinterpreted by many participants and could result in either the inclusion of foods only fed as condiments or the exclusion of foods fed irregularly/ in smaller amounts but which should be included in the recall. Although both open- and closed-ended approaches carry benefits and limitations, findings from this Nepal study highlight that overall the closed-ended approach may result in more accurate estimates.

Many of the cognitive challenges identified in this study among Nepali mothers stem from the linguistic and cultural adaptation of the DHS-8 questionnaire to the Nepal context. First, the translation of “through the night” from English to Nepali resulted in a slightly different interpretation that could lead to differential understandings of the recall periods in the 2 languages. Second, differences in how certain foods are categorized in Nepali culture compared with how these same foods are categorized according to DHS survey designers (for example, which foods are considered *tarkari* (vegetables) and what foods are considered deep-fried compared with sauteed), as well as certain foods not being familiar in the Nepal context (that is, “tinned milk” and various processed meats) also led to cognitive challenges. The impact of language translation and cross-cultural questionnaire adaptation on national survey measurement has been highlighted in prior research using cognitive interviewing. A United States study using cognitive interviews to test the Spanish translation of the National Health Interview Survey found challenges with translation and cultural adaptation, as certain concepts and definitions varied across cultures. [25]. Similarly, another study identified linguistic and culture-specific issues in the Spanish translation of the US Census Bureau’s American Community Survey [26]. Findings from these studies and the present Nepal study emphasize that although commonly accepted methods for verifying translation, such as back-translation and pretesting, are useful approaches, the use of cognitive interviewing may be an additional approach for further verifying linguistic and cultural adaptations of survey tools. Given that the DHS is implemented across 90 countries, all of which have various subnational populations, the use of cognitive interviewing in advance of data collection could serve to identify critical tool adaptation problems and ensure greater measurement accuracy.

Given the relatively new and evolving nature of cognitive interviewing methods in surveys, several reflections on the approach taken for this study are noted. In terms of sample size, conducting 60 cognitive interviews with mothers across 3 districts allowed us to achieve data saturation, as no new information emerged in later interviews and themes became repeated. Probing techniques were effective in enhancing participants' understanding when questions were repeated without additional descriptions. This study used a structured approach to cognitive interviewing, rather than open-ended questioning. Although the latter approach may provide opportunities for participants to bring up cognitive challenges beyond those included in the questionnaire, the structured interview approach allowed for comparison across participants and the ability to focus on particular areas of interest for questionnaire design. Finally, sampling across different SES groups was insightful, as it revealed variations in responses that may have been missed with a homogenous sample. This underscores the value of diverse sampling in capturing a wide range of insights, which can be achieved through sampling based on socioeconomic status as well as other background characteristics including urban/rural residence and ecological zones.

Several limitations of this study should be noted. This was a cross-sectional study so multiple iterations could not be done to assess improvements in cognition with the suggested revisions. Further, there is also a possibility of recall bias in providing information on IYCF practices. Additionally, it should be noted that in the context of actual DHS surveys, respondents are subject to long interviews lasting several hours which can lead to survey fatigue, particularly toward the latter part of the survey where the IYCF module is situated. In this present study, to afford participants more time and mental bandwidth to reflect on answers to the cognitive interview, participants were led through a shorter questionnaire consisting of only sociodemographic questions and the IYCF module. This therefore may not be fully reflective of the typical experience of participants during a complete DHS questionnaire, which may affect the generalizability of our findings. Finally, a purposive sample of participants was used across regions and communities in an attempt to provide a range of SES; however, in the measurement of SES, the majority of participants belonged to high SES groups.

This study identified key cognitive challenges reported by Nepalese participants in answering dietary recall questions in the DHS-8 IYC dietary assessment module. These findings indicate that current measurements may be subject to over or underestimation and there is a need for further research in this area to understand the degree of measurement error related to these challenges. The use of cognitive interviewing for nutrition surveys is recommended to build an understanding of the local interpretation of a survey questionnaire and to ensure effective questionnaire design. Although back-translation and pretesting are considered norms for survey preparation, the inclusion of cognitive interviewing could be an additional component of ensuring data quality that is not substantially cost-prohibitive for large-scale surveys. Although such studies do require additional time and resources, the potential benefit for reliability of indicator measurement may be considerable. These findings can improve IYC dietary assessments conducted by researchers and program implementers using the DHS IYCF dietary assessment module, and aid potential revisions of the IYCF dietary assessment module for future surveys in the country.

## Author contributions

The authors’ responsibilities were as follows – AMP: designed the research; NS, AMP, KY-E: wrote the study protocol; SK, NS: conducted the research and analyzed the data; SK, AMP, KY-E: wrote the article; and all authors: read and approved the final manuscript.

## Conflict of interest

The authors report no conflicts of interest.

## Data Availability

Data described in the manuscript, code book, and analytic code will be made available upon request pending approval.
